# Draft genome sequence of *Streptomyces poriferorum* RTGN2, a bacterial endophyte isolated from *Alnus glutinosa* root nodules

**DOI:** 10.1128/mra.00486-23

**Published:** 2023-12-22

**Authors:** Ryan Michael Thompson, Edward M. Fox, Maria del Carmen Montero-Calasanz

**Affiliations:** 1 School of Natural and Environmental Sciences, Newcastle University, Newcastle upon Tyne, United Kingdom; 2 Department of Applied Sciences, Northumbria University, Newcastle upon Tyne, United Kingdom; 3 IFAPA Las Torres-Andalusian Institute of Agricultural and Fisheries Research and Training, Junta de Andalucía, Cra. Sevilla-Cazalla, Alcalá del Río, Seville, Spain; University of Strathclyde, Glasgow, United Kingdom

**Keywords:** *Streptomyces*, *Alnus glutinosa*, genomes, endophytes, actinobacteria, root, nodule

## Abstract

Herein is reported the draft genome sequence of *Streptomyces poriferum* RTGN2, a bacterial isolate of *Alnus glutinosa* root nodules, collected from Saltwell Park, Gateshead, United Kingdom. The assembly is 9.5 Mbp in size, composed of 187 contigs, with a N_50_ of 189,630 bp, presenting a GC content of 71.2%.

## ANNOUNCEMENT

The bioremediative abilities of *Alnus glutinosa* have led to increased studies of these trees; however, there is a lack of knowledge regarding the tree’s root nodules, with majority of current knowledge focused upon their principal endosymbiont *Frankiaceae* (1–3). As such, bacterial isolation was attempted from *A. glutinosa* nodules to study non-*Frankiaceae* inhabitants.

*Streptomyces poriforum* RTGN2 was isolated from *A. glutinosa* nodules, collected from a single tree within Saltwell Park, Gateshead, United Kingdom (54.944723–1.605852). The nodules were washed under tap water to remove soil, then 4–15 nodule lobes were cut from the larger nodule mass and surface sterilized using 1 mL of 25% strength household bleach (~1.125% sodium hypochlorite) by agitating the tube for 5 minutes. The bleach was discarded and the nodule lobes were washed five times in 1 mL of sterile distilled water for 5 minutes, after which they were homogenized in 0.5 mL of 1/4-strength Ringers solution, with a further 14.5 mL being added and this being plated upon nitrogen-free basic propionate agar (50-mg/L nystatin) plates and incubated at 28°C for 8 days (https://www.dsmz.de/microorganisms/medium/pdf/DSMZ_Medium1536.pdf) (4). Strain RTGN2 was recovered by cultivating a single colony onto fresh nitrogen-free basic propionate agar for 5–7 days at 28°C. Three iterations of subculturing were conducted from a single colony to achieve an axenic culture, with material being stored in 30% glycerol at −80°C.

RTGN2 was cultivated upon GYM agar for 3 days (https://www.dsmz.de/microorganisms/medium/pdf/DSMZ_Medium65.pdf), after which DNA was extracted using a GenEluteBacterial Genomic DNA Kit (Sigma-Aldrich, USA) and sequenced by Novogene Co. Ltd. The DNA library was prepared using the Novogene NGS DNA library prep set (Cat No.PT004) in which the DNA was randomly sheared into short fragments, end repaired, A-tailed, then ligated with the Illumina adaptor. These sequences were amplified using PCR, size selected for 350 bp, purified, and sequenced using 150-bp Illumina paired-end sequencing upon the Illumina NovaSeq platform.

In the following analysis, default settings were used unless otherwise noted. The 12,931,706 reads generated from Illumina sequencing of RTGN2 were filtered using FastP (v.0.23.1) (5) to remove adapter sequences, reads that contained more than 10% uncertain nucleotides, or more than 50% low-quality reads (base quality <5). This filtering left 99.7% of the reads, of which 96.4% were deemed Q_20_, and 91.1% of these were deemed Q_30_. The filtered reads were uploaded to Galaxy Europe (https://usegalaxy.eu/) (6) and assessed using FASTQC (Galaxy v.0.73 + galaxy0) (http://www.bioinformatics.babraham.ac.uk/projects/fastqc/), assembled using Shovill (Galaxy v.1.1.0 + galaxy1) (https://github.com/tseemann/shovill), with the contig quality assessed using Quast (Galaxy v.5.0.2 + galaxy5) (7–9). Of these contigs, 18 were removed as they were below 200 bp in length. The relationship of RTGN2 to its nearest neighbor *Streptomyces poriferum* P01-B04T was determined using the TYGS webserver (v.389), with a 40.9% dDDH similarity noted (10, 11).

The assembled genome was 9,472,222 bp in length, composed of 187 contigs, which displayed an N_50_ of 189,630 bp and a GC% of 71.15%. Based upon the Shovill output, the estimated sequencing depth was 205×.

## Data Availability

The draft genome and Sequence Read Archive data of Streptomyces RTGN2 were deposited in International Nucleotide Sequence Database Collaboration under the accession numbers JAPZLE000000000 and SRR22064603, respectively.
